# SARS-CoV-2 surveillance with environmental surface sampling in public areas

**DOI:** 10.1371/journal.pone.0278061

**Published:** 2022-11-23

**Authors:** Kristina Mihajlovski, Mark P. Buttner, Patricia Cruz, Brian Labus, Barbara St. Pierre Schneider, Elizabeth Detrick

**Affiliations:** 1 Department of Environmental and Occupational Health, School of Public Health, University of Nevada, Las Vegas, NV, United States of America; 2 Graduate Nursing Department, College of Nursing and Health Innovation, The University of Texas at Arlington, TX, United States of America; University of Helsinki: Helsingin Yliopisto, FINLAND

## Abstract

Contaminated surfaces are one of the ways that coronavirus disease 2019 (COVID-19) may be transmitted. SARS-CoV-2 can be detected on environmental surfaces; however, few environmental sampling studies have been conducted in nonclinical settings. The objective of this study was to detect SARS-CoV-2 RNA on environmental surfaces in public areas in Las Vegas, Nevada. In total, 300 surface samples were collected from high-touch surfaces from high-congregate public locations and from a public health facility (PHF) that was visited by COVID-19 patients. Environmental samples were analyzed with quantitative reverse-transcriptase polymerase chain reaction (RT-qPCR) using SARS-CoV-2 specific primers and probes for three target genes. Results showed that 31 out of 300 (10.3%) surface samples tested positive for SARS-CoV-2, 24 at the PHF and 7 in high-congregate public locations. Concentrations ranged from 10^2^ to 10^6^ viral particles per 3 ml sample on a wide variety of materials. The data also showed that the N gene assay had greater sensitivity compared to the S and ORF gene assays. Besides frequently touched surfaces, SARS-CoV-2 was detected in restrooms, on floors and surfaces in contact with floors, as well as in a mop water sample. The results of this study describe the extent and distribution of environmental SARS-CoV-2 contamination in public areas in Las Vegas, Nevada. A method using the N gene PCR assay was developed for SARS-CoV-2 environmental monitoring in public areas. Environmental monitoring with this method can determine the specific sites of surface contamination in the community and may be beneficial for prevention of COVID-19 indirect transmission, and evaluation and improvement of infection control practices in public areas, public health facilities, universities, and businesses.

## 1. Introduction

In December 2019, the world faced an outbreak of a new emerging pathogen called SARS-CoV-2. This new virus was first detected in the city of Wuhan, China, and was found to cause coronavirus disease 2019 (COVID-19) [1]. In January 2020, the World Health Organization (WHO) characterized this outbreak as a public health emergency of international concern, resulting in the WHO’s highest level of alarm [2]. On March 11, 2020, the WHO declared COVID-19 as a pandemic [3]. COVID-19 is transmitted from person to person via respiratory droplets, through airborne spread, from contact with animals, and contact with contaminated surfaces (fomites) [4].

Indirect transmission of COVID-19 occurs when an infected patient’s respiratory droplets or secretions contaminate surfaces that are later being touched by a healthy individual. Once a healthy person touches their eyes, nose, or mouth, a COVID-19 infection may occur [5]. Fomites were found to be a mode of SARS-CoV-2 transmission in an animal study model [6]. Compared to aerosol and intranasal exposure to SARS-CoV-2, fomite exposure resulted in milder clinical presentation among study animals. Environment-to-human transmission was researched in a mathematical modeling study conducted by Wang et al. [7], where it was concluded that hand hygiene and surface disinfection are among crucial public health measures for COVID-19 prevention.

Marcenac et al. [8] detected SARS-CoV-2 RNA on nightstands, pillows, and light switches in COVID-19 positive households. Viable virus was isolated only from one nightstand sample [8]. Piana et al. [9] concluded that fomites are significant indicators of SARS-CoV-2 indirect transmission, as respiratory droplets and biofluids were detected on them. The authors collected 92 surface samples from hospitals, public buildings, and outdoor spaces, and found that SARS-CoV-2 was detected only in COVID-19 patient’s surrounding. Their findings are emphasizing the importance of hand hygiene and environmental disinfection.

SARS-CoV-2 may be detected on surfaces comprised of different materials, such as plastic, stainless steel, cardboard, copper, paper, surgical masks, and human skin, for up to several days [10–12]. Riddell et al. [13] found that SARS-CoV-2 stability on different surfaces depends on temperature and relative humidity (RH). They found that this virus can remain viable on stainless steel at room temperature (20°C, 50% relative humidity) for more than 28 days. Biryukov et al. [14] conducted an experimental study that found that COVID-19 fomite transmission may occur for hours and days in an indoor setting. They detected SARS-CoV-2 on stainless steel, plastic, and rubber glove coupons, and found that with increased temperature and RH, the half-life of SARS-CoV-2 present on surfaces decreased. Ashghar et al. [15] also concluded that contaminated surfaces may be one of the significant routes of COVID-19 transmission.

In a patient care setting, Chia et al. [11] detected SARS-CoV-2 on high-touch surfaces in patient isolation rooms during and after their first week of disease. Most commonly contaminated surfaces were personal protective equipment (PPE), medical equipment, and sanitizer dispensers [16]. Wei et al. [17] found environmental SARS-CoV-2 contamination in COVID-19 asymptomatic patients’ surroundings. In a nonclinical, workplace setting, Marshall et al. [18] found that workplaces with a significant prevalence of SARS-CoV-2 environmental samples had 10 times greater chances of having COVID-19 positive employees, compared to the places with negative environmental samples. Break room door handles, faucets, workbenches, and break room chairs were the predominant surfaces of SARS-CoV-2 detection [18]. Moreover, these results indicate that the presence of asymptomatic COVID-19 cases may be detected by environmental monitoring of workplaces.

Other researchers have focused on environmental surfaces in public locations. Harvey et al. [19] collected environmental surface samples from 33 different locations in Massachusetts including a gas station, laundromat, retail businesses, and surfaces, such as a trash can, a metro entrance, and a post office box. Results showed that 29 of 348 (8.3%) of environmental surface samples were SARS-CoV-2 positive. Because low levels of SARS-CoV-2 contamination was detected on public surfaces, the authors estimated that COVID-19 infection from these surfaces is possible, but with low risk. Cai et al. [20] reported a correlation between COVID-19 spread in a shopping mall in China and contaminated surfaces. Cases reported no close contact to other COVID-19 cases from the mall; however, they all used the same locations within the shopping mall (i.e., restrooms and elevators). During a COVID-19 outbreak on a cruise ship, Yamagishi et al. [21] conducted environmental sampling in the cabins of COVID-19 positive passengers between Day 1 and Day 17 after COVID-19-positive passengers disembarked. SARS-CoV-2 RNA was detected in 58 of 601 (9.7%) of environmental samples, collected in both symptomatic and asymptomatic case cabins.

Since the COVID-19 pandemic began, environmental monitoring has emerged as an important component of public health surveillance [22]. Wastewater surveillance has been used to assess the presence and prevalence of SARS-CoV-2 circulating in communities [23]. Surface sampling may be useful in combination with wastewater surveillance to pinpoint the specific locations of pathogens in a community. According to the WHO, to gain more knowledge about COVID-19, environmental surveillance should be one of the main public health goals [24]. However, COVID-19 environmental sampling studies are lacking, particularly at congregated, international travel destinations. There is a need for further research to determine the extent of contamination of public areas with SARS-CoV-2. The objective of this study was to detect SARS-CoV-2 RNA in environmental surface samples in selected public areas in Las Vegas, Nevada.

## 2. Materials and methods

### 2.1. Environmental surface sampling

Environmental surface sampling was conducted in high-traffic public locations and at a PHF in Las Vegas, Nevada from December 7, 2020 until April 22, 2021. In total, 300 environmental surface samples were collected from these locations; 150 samples were collected from high-congregate public locations, and 150 samples were collected from a PHF that was visited by COVID-19 patients. Permission for field site access and sampling at the PHF was obtained from Southern Nevada Health District. High-congregate public locations samples were collected from frequently touched surfaces in high-traffic places, such as gas stations, crosswalk pedestrian push buttons, post office, car washes, restrooms in grocery stores and shopping malls (Table 1). PHF samples were collected from surfaces frequently touched by the staff and COVID-19 patients, such as door locks, faucets, tables, chairs, and copy machines. Information about types of sampled surface materials was recorded.

**Table 1:** Sampling locations.

Sampling Locations (number of samples)
Condominiums (4)
Bus Station (1)
Hardware Stores Restrooms (16)
Grocery Stores Restrooms (28)
Las Vegas Boulevard Escalators (4)
Las Vegas Boulevard Elevators (3)
Crosswalk Pedestrian Push Buttons (3)
Retail Store Restrooms (12)
Retail Store Carts (2)
Post Office (1)
Water Mill (4)
Carwash (2)
Gas Stations (10)
Department Store Restroom (1)
Casino Parking (3)
Bus Pass Machine (1)
University Library (20)
University Restrooms (9)
University Locations (6)
Shopping Mall Restrooms (20)
Public Health Facility Site 1 (10)
Public Health Facility Site 2 (140)

Surface environmental sampling was conducted with sterile foam tipped applicators (Puritan, Guilford, ME) that were immersed in 3 ml viral transport medium (VTM) (Hardy Diagnostics, Santa Maria, CA). Sample collection kits were stored at 4°C until ready to be used. Prelabeled kits were transported on ice in a designated, biohazard labeled cooler to the sampling location. The sampling was conducted with moistened swabs in an overlapping pattern, according to CDC sampling protocols designed for the collection of bacterial spores as environmental biocontaminants [25]. An area of approximately 26 cm^2^ was sampled. After surface sampling, the exposed swabs were returned to the VTM. All sampled surfaces were disinfected with an isopropyl alcohol wipe after sample collection. After environmental surface sampling, the samples were placed in the cooler, transported to the laboratory within 24 h, and stored at -20°C until RNA extraction and PCR analysis.

### 2.2. SARS-CoV-2 RNA extraction

To isolate SARS-CoV-2 RNA from surface samples, RNA extraction was performed using the QIAamp DSP Viral RNA extraction kit (Qiagen, Hilden, Germany), according to the manufacturer’s protocol. A volume of 420 μL of each sample in VTM was extracted. The final extract volume of 60 μl was stored at -70°C until RT-qPCR analysis.

### 2.3. Quantitative Reverse Transcription Polymerase Chain Reaction (RT-qPCR) analysis

An RT-qPCR method authorized for COVID-19 testing was used [26]. RT-qPCR analysis of samples was conducted with the QuantStudio™ 6 Pro Real-Time qPCR instrument (ThermoFisher Scientific, Waltham, MA), using the SARS-CoV-2 N PCR assay kit. (ThermoFisher). In this study, the TaqMan™ 2019nCoV Assay Kit v1 (Catalog Number A47532) was used for detection of SARS-CoV-2 in environmental samples. A one-step RT- qPCR Master Mix and an internal positive control (IPC) (ThermoFisher) were used. Each PCR reaction had 25μl of total volume that consisted of 5 μl sample extract, 9.5 μl ultrapure water, 1.25 μl N gene assay (i.e., primers and probes), 6.25 μl 4X TaqMan® PCR Master Mix, 2.5 μl 10X IPC Mix, and 0.5 μl 50X IPC DNA. Amplification was conducted in standard mode, with the following conditions: 25°C for 2 min, 50°C for 15 min, 95°C for 2 min, and 40 cycles of 95°C for 3 sec followed by 60°C for 30 sec. All samples were amplified in duplicate. A non-template control (NTC) that contained nuclease free water, and a positive control (SARS-CoV-2 RNA, ThermoFisher) were included in each PCR analysis.

In this study, the SARS-CoV-2 N gene assay was used as it is more conserved and stable, compared to the S and ORF genes ([27–29]. A TaqMan® internal positive control (IPC) (ThermoFisher), using a VIC^TM^ /TAMRA probe was used to determine if there was PCR inhibition in the environmental samples. Serial dilutions of a standard with a known SARS-CoV-2 RNA concentration (1 x 10^4^ copies/μl; ThermoFisher) were analyzed in duplicate, along with environmental samples. The instrument software created a standard curve of cycle threshold (Ct) values, which was used to confirm the presence and concentration of SARS-CoV-2 RNA in the environmental samples. The PCR Ct values of these standards were used to calculate the number of viral particles per sample.

Mean Ct values of duplicate samples were used to analyze the amplification results. RT-qPCR data were entered into a spreadsheet to determine the number and location of positive samples, as well as the number of SARS-CoV-2 RNA copies per sample. Positive samples were verified by additional RT-qPCR analyses using the S and ORF gene markers (ThermoFisher). Negative samples were analyzed using an IPC PCR to rule out false negative results due to environmental inhibition. For assay comparison, S and ORF gene RT-qPCR tests were conducted. The amplification conditions for S and ORF gene RT-qPCR assays were the same as for the N gene assay.

## 3. Results

### 3.1. Environmental surface sample analysis

In this study, 31 out of the 300 (10.3%) environmental surface samples tested SARS-CoV-2 RNA positive (Table 2). Sixteen percent (24 of 150) of the PHF samples tested positive. In addition, 7 out of 150 (4.7%) samples collected in high-congregate public locations tested positive. Positive samples from these locations included a door handle, a handrail, elevator buttons, gas station pumps, crosswalk pedestrian push button, and restroom toilet seats, sinks and doorknobs. No PCR inhibition was observed in these samples.

**Table 2:** SARS-CoV-2 positive samples and RNA copies per sample.

Sample Name	Number of viral RNA copies per sample
Door Locks in Male Restroom	2.26E+03
Las Vegas Boulevard Elevator 2 Buttons	2.79E+03
PHF Site 2 Clothes Locker	6.97E+02
PHF Site 2 Restrooms-Faucets and Flush Buttons	6.03E+04
PHF Site 2 Restrooms- Sinks	7.78E+06
PHF Site 2 Coffee Table 1	2.74E+05
PHF Site 2 Coffee Table 2	8.09E+04
PHF Site 2 Couch 2	1.06E+04
PHF Site 2 Table 1	1.81E+05
PHF Site 2 Restroom 2- Toilet Seat	5.37E+03
PHF Site 2 Restroom 1- Area Around Toilet	2.60E+04
PHF Site 2 Dining Table 3	3.38E+05
PHF Site 2 Front Desk and Pen	2.17E+03
PHF Site 2 Copy Machine	3.46E+03
PHF Site 2 Restroom 2- Area Around Toilet	3.93E+06
PHF Site 2 Chairs 3	4.33E+03
PHF Site 2 Security Team Radio	6.90E+03
PHF Site 2 Medical Staff Shoes	1.03E+04
PHF Site 2 Security Staff Shoes	5.33E+03
PHF Site 2- Researchers’ Shoes	2.47E+05
PHF Site 2 Mop Water	5.05E+04
PHF Site 2 Linen Carts Wheels	1.18E+04
PHF Site 2 Cleaning Station 1 Wheels	1.54E+05
PHF Site 2 Cleaning Station 2 Wheels	2.95E+04
PHF Site 2 Medical Staff Chair Wheels	2.71E+04
PHF Site 2 Oxygen Tank Wheels	2.17E+04
University Library Female Restroom Sinks	1.77E+04
University Facility Male and Female Restroom Door Knob	2.08E+03
Crosswalk Pedestrian Push Buttons	7.71E+03
Gas Station- Gas Pump Buttons, Credit Card Pin Pad Buttons, Gas Selection Buttons AND Keypad of Condominium	7.85E+03
Grocery Store Metal Trash Can in Female Restroom	7.15E+03

The Ct values of positive samples ranged from 25.8 to 38.4. Quantification was conducted with the N gene target only, as this target showed the highest detection rate in this study. The PCR software calculated the standard curve plot (r^2^ = 0.984) with the Y-intercept (41.252) and slope (-3.182) values. Based on the standard curve equation and mean Ct values of environmental samples, the concentrations of SARS-CoV-2 RNA copies per 3 ml sample were calculated (Table 2). The lowest number of SARS-CoV-2 RNA copies detected was 697 per sample, which was obtained from the PHF Site 2 Clothes Locker sample. As the locker was located outdoors, the temperature might have contributed to the decreased RNA concentration on this surface. The highest number of SARS-CoV-2 RNA copies was 7.8 x 10^6^ per sample and found in the PHF Site 2 Restroom Sink sample. A sample that also had a high concentration of RNA (3.9 x 10^6^ RNA copies) was the PHF Site 2 Restroom-Floor Area Around Toilet. This finding indicates that SARS-CoV-2 RNA concentrations are increased in areas where patient’s respiratory fluids and secretions are present.

### 3.2. Surface materials

The SARS-CoV-2 positive samples were found on rubber (8), plastic (8), stainless steel (6), vinyl (2), wood (2), ceramic (2), metal (1), artificial leather (1), and in the mop water (1). The virus was detected most often on rubber (8 of 300 samples), plastic (8 of 300 samples), and stainless-steel surfaces (6 of 300 samples); however, these surfaces were sampled the most frequently. In the PHF, all floor samples, and samples of objects in contact with the floor (e.g., chair wheels, cart wheels, and staff’s shoes) tested positive. A positive mop water sample was collected from a mopping bucket that was used for floor cleaning. This finding indicates that SARS-CoV-2 viral RNA can be found in floor-cleaning water that contains detergent.

### 3.3. N, S, and ORF gene assays

For comparing the N gene PCR assay with the S and ORF assays, 10 positive samples were selected that had the strongest signal (lowest mean Ct values). Both S and ORF gene assays showed positive results for samples that were positive with the N gene assay. The lowest mean Ct value was observed with the N gene assay (mean Ct = 27.11 ± 0.72 [SE]), followed by the S gene assay (mean Ct = 29.31 ± 0.55), whereas the highest Ct value (the weakest signal) was observed with the ORF gene assay (mean Ct = 29.86 ± 0.60). Statistical analysis with a one- way analysis of variance revealed that there was a significant difference between the mean Cts of the three gene assays (p = 0.005), indicating that the N gene assay had greater sensitivity compared to the S and ORF gene assays.

### 3.4. Assay sensitivity

The limits of SARS-CoV-2 RNA detection in environmental surface samples were calculated. Assuming the detection of 1–10 RNA copies in the PCR reaction, which was determined by the standard curve, sensitivities of the assays were calculated based on the sample volume, the fraction of the sample processed for RNA extraction, and the amount of RNA extract used in the PCR assay. The limits of detection were between 86 and 860 RNA copies per sample, or approximately 3 to 33 RNA copies per cm^2^.

## 4. Discussion

Environmental surface surveillance is a useful public health tool that has been used for detection and mitigation of various infectious diseases. In their environmental surface sampling study, Zhang et al. [30] detected SARS-CoV-2 on university campus surfaces, indicating that routine and multidisciplinary environmental surveillance approaches should be used. Cheng et al. [31] sampled 377 environmental surfaces in a hospital, and found a 5% SARS-CoV-2 positivity rate. Harvey et al. [19] found an 8.3% SARS-CoV-2 positivity rate among their 348 environmental surface samples. In this study, of 300 environmental surface samples, the positivity rate was 10.3%.

### 4.1. Materials

In this study, SARS-CoV-2 was detected on numerous frequently touched environmental surfaces in high-congregate public locations and at a PHF in Las Vegas, Nevada, including plastic, stainless steel, rubber, metal, and vinyl. In addition, rubber, plastic, and stainless steel were the materials on which SARS-CoV-2 was detected most frequently. This finding is supported by van Doremalen et al. [10] who also detected SARS-CoV-2 on stainless steel and plastic, and found that this virus remained the longest on these two materials. Under experimental conditions, Liu et al. [32] found that SARS-CoV-2 was detected on stainless steel, plastic, ceramic, and wood, and remained viable for 7 days. Cantu et al. [33] found SARS-CoV-2 RNA in the bathroom sink, on the bedside table, kitchen table and sink handles of COVID-19 patients isolation apartments. The authors concluded that surface sampling may be used for early detection of COVID-19 in a school setting, as well as in prisons and group living, where viral shedding would be difficult to detect with other monitoring measures. In our study, rubber was another material on which SARS-CoV-2 was detected the most, predominantly at the PHF setting. Moreover, a study conducted by Paton et al. [34] found that SARS-CoV-2 remained viable on stainless steel for 114 h. Abrahão et al. [35] conducted a study in Brazil and found SARS-CoV-2 on metal, plastic, wood, as well as on the floor of a bus station. In our study, SARS-CoV-2 was found on all these surfaces; however, the viability of the virus was not determined. According to CDC data, the risk of SARS-CoV-2 transmission from surfaces is less than 1 in 10,000 [36]. However, the contribution that SARS-CoV-2 surface contamination has on COVID-19 transmission is unknown.

### 4.2. SARS-CoV-2 on floor surfaces

Results showed that all the floor samples collected at the PHF, as well as samples of the objects that were in contact with the floor tested positive for SARS-CoV-2, including staff’s shoes. This finding is supported by the results of Guo et al. [37] who detected SARS-CoV-2 on half of the samples from medical staff shoes. They also found that 7 out of 10 intensive care unit (ICU) floor samples tested positive. Renninger et al. [38] conducted a study at the healthcare facility, and detected the presence of SARS-CoV-2 RNA in the floor dust samples in COVID-19 patient’s rooms. Floor samples continued to test positive for four weeks, despite the floor disinfection practices. This is consistent with the finding of our study, where SARS-CoV-2 was detected in the mop water bucket that contained water and detergent for PHF’s floor cleaning. This indicates the potential for viral particles to be spread from contaminated floors, and to be detected in the water with the disinfectant solution. However, the infectivity of the virus in the mop water is unknown. Future wastewater surveillance studies may be conducted to understand if SARS-CoV-2 may remain infectious in the detergent or disinfectant containing water.

### 4.3. SARS-CoV-2 in restrooms

The PHF restrooms used by COVID-19 patients and public restrooms in Las Vegas were locations where SARS-CoV-2 was detected in the highest concentrations in this study. Several studies in the clinical setting showed that samples collected from patients’ restrooms, such as samples from toilet seats, floors, and sinks, tested positive for SARS-CoV-2 [21,39]. A study by Dancer et al. [40] found that SARS-CoV-2 was detected on numerous public restroom surfaces, including sinks. Rajendiran et al. [41] found an increased SARS-CoV-2 detection rate in the patient restroom area, compared to patients’ rooms. They detected the virus on sinks and toilet bowl. In the current study, the highest number of SARS-CoV-2 RNA copies was detected in patient restroom sinks in the PHF. This may indicate that a high number of viral particles in respiratory and/or oral excreta from COVID-19-positive patients may be deposited on sink surfaces. Urine, feces, oropharyngeal, and nasal secretions are potential restroom SARS-CoV-2 sources, which is currently being confirmed by wastewater surveillance. Restroom samples from this study had the highest viral RNA load, which confirms findings of other studies, and indicates the need for wastewater surveillance. Zhao et al. [42] concluded that the high viral load of SARS-CoV-2 particles found in feces, urine, nasal and oropharyngeal secretions is beneficial for wastewater surveillance. Peng et al. [43] detected viral RNA in human urine, and Liu et al. [32] found that SARS-CoV-2 can remain viable for a few hours in human feces, and for three to four days in human urine samples.

### 4.4. Frequently touched surfaces

The PHF in which samples were collected was a facility designated for COVID-19 patients. Results indicated that surfaces frequently touched by patients and staff tested positive for SARS-CoV-2. This finding is supported by the study of Ben-Shmuel et al. [44] who found that surfaces frequently touched by patients and medical staff in two hospitals tested positive. A hospital environment study conducted by Ye et al. [16] found that objects that were most often contaminated with SARS-CoV-2 in a medical center in Wuhan, China were printers, desktop/keyboard, and door knobs frequently touched by patients and medical professionals. This finding is in agreement with the results of this study, which found that a copy machine, keyboard/mouse, and doorknobs at the PHF tested positive.

### 4.5. PCR assay sensitivity

SARS-CoV-2 has four structural proteins: the spike (S) protein, membrane (M) protein, envelope (E), and nucleocapsid (N) protein [45]. The S, M, and E proteins form the viral coat, whereas the N protein helps packaging of RNA and genome protection. The S protein is a glycoprotein of the viral envelope that helps viral attachment and entry into host cells [46]. In addition, the N gene is highly conserved [29]. Moreover, the SARS-CoV-2 genome contains 10 open reading frames (ORF) genes which code for non-structural proteins that are not essential for RNA replication. All these gene targets are currently used to detect SARS-CoV-2 in clinical and environmental samples. As new SARS-CoV-2 variants continue to emerge, it is important to determine which gene targets are the most sensitive for detection of the virus.

This study demonstrated that the N gene PCR assay was more sensitive compared to the S and ORF assays, respectively. However, as genomic sequencing of viral RNA was not conducted in samples, it is unknown whether SARS-CoV-2 variants were also detected. According to Naqvi et al. [29], the N gene is a highly conserved genetic region. However, Wang et al. [47] found that the N gene is one of the least conserved genes. Tahan et al. [48] discovered that the S gene and E gene assays may not detect SARS-CoV-2 variants, due to mutations in those gene regions. Therefore, multiple gene target PCR tests may be needed to improve the overall detection of SARS-CoV-2. However, based on the results of this study, the N gene PCR assay may be used for the environmental surface sampling studies.

### 4.6. Sample quantification

Quantification of SARS-CoV-2 RNA was conducted to estimate the viral load present in each 3 ml sample and the sensitivity of the assay. Cardinale et al. [49] found that SARS-CoV-2 concentration in environmental samples analyzed by their droplet detection RT-PCR method ranged from 5.6 to 132 RNA copies per cm^2^, with no viral infectivity analysis. The sensitivity of the assay in the current study was comparable, ranging from 3 to 33 copies per cm^2^. In this study, viability of SARS-CoV-2 virus collected from environmental samples was not determined; therefore, the potential for indirect transmission is unknown.

Wang et al. [7] assumed that high environmental SARS-CoV-2 concentration leads to increased viral load in infected individuals, resulting in severe clinical presentation. Based on their mathematical simulation, environmental surface transmission significantly contributed to the detection of symptomatic and pre-symptomatic COVID-19 cases. Moreover, the authors concluded that COVID-19 transmission cannot be prevented if environmental transmission is not controlled. In our study, we detected the highest levels of SARS-CoV-2 RNA on surfaces that were touched by SARS-CoV-2 symptomatic patients.

### 4.7. Study limitations

This study was limited by a 4-month sample collection time frame that encompassed variable environmental conditions and infection rates in the community. A positive RT-qPCR analysis could not confirm whether the virus detected was viable and had potential to cause an infection. While the swab sampling method proved to be successful in the detection of SARS-CoV-2, the collection efficiency of method is unknown. The sampling area of 26 cm^2^ was an approximation as some swab samples were used to collect samples from multiple surfaces (i.e., composite sample of gas station buttons and pin pads). In addition, as the virus mutates, it cannot be stated with certainty whether SARS-CoV-2 variants were detected.

## 5. Conclusion

The results of this study describe the extent and distribution of environmental SARS-CoV-2 contamination in frequently touched, high-congregate public locations and in a public health facility in Las Vegas, Nevada. A method using the N gene PCR assay was developed for SARS-CoV-2 environmental monitoring in public areas. In addition, environmental surveillance of frequently touched surfaces, particularly restrooms and floor surfaces in combination with wastewater surveillance may be an additional tool for more specific detection of viral shedding sites in the community and high- congregate settings. Moreover, environmental surveillance may help in detection of asymptomatic COVID-19 cases, and therefore improve outbreak control, particularly in high-congregate settings where other outbreak mitigation measures may not be achievable. Although the viability and infectivity of the viral particles on these surfaces were not determined, the high copies of viral particles detected on various surfaces in public restrooms may warrant provisions for hand washing policies, frequent custodial restroom cleaning and engineering controls within restrooms that limit hand touching of surfaces (e.g., automatic water, soap, and paper towel dispensers). Environmental surface surveillance, along with wastewater surveillance, contact tracing, case investigation, and other epidemiological measures can be utilized as a useful tool for SARS-CoV-2 surveillance and mitigation. Environmental monitoring with the method developed in this study can determine the specific sites of surface contamination in the community and may be beneficial for prevention of COVID-19 indirect transmission, and evaluation and improvement of infection control practices in public areas, public health facilities, universities, and businesses.
